# Editorial: Underlying neurobiological, genetic, and behavioral mechanisms in schizophrenia and autism spectrum disorder

**DOI:** 10.3389/fpsyt.2025.1694809

**Published:** 2025-09-29

**Authors:** Silvia Corbera, Rafael Penadés, Michal Assaf

**Affiliations:** ^1^ Department of Psychological Science, Central Connecticut State University, New Britain, CT, United States; ^2^ Hospital Clinic of Barcelona, Barcelona, Spain; ^3^ Olin Neuropsychiatry Research Center and Mary W. Parker Autism Center, Institute of Living, Hartford Hospital, Hartford, CT, United States; ^4^ Department of Psychiatry, Yale School of Medicine, New Haven, CT, United States

**Keywords:** schizophrenia, autism spectrum disorder, neurobiological, transdiagnostic, genetic, behavioral mechanism

Understanding potential joint underlying mechanisms of Schizophrenia (SZ) and Autism Spectrum Disorder (AT) is an essential area of study given their phenotypic overlap, including in social communication and social cognition differences (1). This Research Topic sought to invite contributions from researchers to share new scientific evidence on the common and distinct phenotypes and underlying mechanisms between and within SZand AT by employing diverse clinical, behavioral, and neuroscience techniques (e.g., neuroimaging, electrophysiology, genetics, symptom characterization, neurocognitive assessments). The overarching goal of this topic of research is to shed light on the functional significance of symptom profiles and find routes to common new venues for interventions in AT and SZ. The studies shown in this Research Topic reflect the growth of the current scientific landscape, illustrating the broadening of the standard techniques to new territories and their aim to discover possible common mechanistic pathways.

While research directly comparing AT and SZ is not common, few contributions to this topic compared their clinical and cognitive profiles. Nakamura et al. aimed to identify clinical similarities and differences in AT and SZ and proposed a new useful objective predictive model that could aid with differential diagnosis. For that purpose, the authors used a combination of items from assessments validated and typically used in one of the groups only, including the Autism Diagnostic observation Schedule, 2^nd^ edition (ADOS-2) and the Positive and Negative Syndrome Scale (PANSS) to develop a predictive diagnostic model. Their studies revealed that negative symptoms in SZ elevated the ADOS-2 ratings and the ADOS-2 false-positive rates of SZ. Therefore, their proposed predictive model to distinguish SZ and AT included a combination of specific ADOS-2 items (A7, A10, B1, B6, B8, and B9). The study of Nakamura et al. echoed the findings from previous studies by Trevisan et al. (2) and Corbera et al. (3), highlighting the need for better, cross-diagnostic symptom measures for differential diagnosis, given the heterogeneity in clinical presentation of both disorders. Other studies have attempted to examine the shared dysfunctions in these disorders by focusing on neurocognitive profiles instead of symptom profiles. For example, Morais et al. examined the executive functioning (EF) profiles in AT, SZ and neurotypical (NT) individuals. Their outcomes showed a substantial overlap in EF deficits with only a few differences in working memory; thus, they suggested tailoring cognitive remediation interventions to these deficits in both populations. While the sample size in this study is relatively small (n=15/group), and results should be replicated, it supports similar interventions for both AT and SZ. Complementing this, in their review, Guerrera et al. advocated for a neurodevelopmental perspective when examining SZ and AT, encouraging the development of large longitudinal studies comparing their phenotypic profile from childhood.

Individual studies into disorder-specific mechanisms can also provide valuable insights. For example, the study by Chen et al. investigated social-emotional interference in AT children with IQ>80 using an experimental design with modified flanker tasks. Their study provided insightful information about emotional processing in autistic children as they showed that they struggle more than typically developing controls in controlling interference from social information, and that they faced more difficulties as the information load increased, which could hinder their ability to adapt to and engage in social situations. The authors proposed practical, translational strategies offering direct implications for intervention in complex social scenarios such as providing a gradual exposure transition from virtual faces to real faces, and from single faces to different faces. Even though this study focused solely on AT, it provided a potential early biomarker to target for future studies potentially applicable to SZ, and warranting future cross-disorder research.

While genetic studies examining the risk of autism have grown over the years, fewer have examined the interaction between genetic and environmental risk factors. The study by Zhang et al. focused on examining telomere length (TL), considered to be a biomarker of cumulative intracellular oxidative stress (OS) in autism. This study showed a relationship between TL shortening and oxidative damage in AT and underscores the potential value of measuring OS-related biomarkers in autism for early diagnosis and interventions. Therefore, comparing the role of OS imbalance in the pathophysiology of AT and SZ is an additional area of further research and a promising area for the generation of treatment interventions.

Studying genomic information involves the use of large-scale, well-powered studies. In this regard, Mas et al. proposed the development of a large-scale multicenter study involved in the development and validation of predictive algorithms to classify first-episode psychosis (FEP) patients according to their response to antipsychotics, to select the most appropriate treatment strategy for each patient. In this large protocol, Mas et al. propose to employ machine learning techniques to integrate predictors, such as pharmacogenetic, epigenetic, and clinical data, and sociodemographic, environmental, and neuroanatomical data, to enhance personalized medicine. This proposed protocol highlights the importance of the interplay of pharmacogenomic studies and precision medicine as a translational step to applicability to clinical practice, an area without doubt, of scarcity in the comparison of SZ and AT.

Among the innovative contributions to this topic, the study by Li et al. performed a systematic search of 30 clinical studies aimed to examine the gut microbiota in patients with SZ, as previous reports had shown changes in the composition of the gut microbiota in mental conditions. They reported a consistent dysbiosis of microbiota in SZ and a large heterogeneity of SZ in these measurements. There is a paucity of research in this area, especially in comparative studies examining the gut-brain axis and the role of alterations in the gut microbiota in the pathogenesis of SZ and AT, which we encourage future researchers to pursue. Finally, Ni et al. aimed at examining the causal link between chronic obstructive pulmonary disease (COPD) risk and SZ using trans-ethnic Mendelian randomization analyses, employing data from public genome-wide association studies (GWAS) datasets. The authors found a causal relationship but mediated by variables such as body mass index (BMI), smoking, and major depressive disorder, only in individuals with European ancestry. Additionally, Ni et al. raised a concern about the scarcity of studies on this topic in autism. Given the conflicting evidence in the prevalence of asthma in this population, and the common vulnerabilities between SZ and AT, this is another area where further research can explore mediators of the link to COPD, in larger scale GWAS studies, and with diverse ancestral populations.

Looking ahead, future research should continue to prioritize transdiagnostic approaches that examine shared and distinct mechanisms across SZ and AT, with a focus on integrative models that combine genetic, environmental, neurobiological, and behavioral data. Large-scale, longitudinal studies are essential to disentangle developmental trajectories and identify early biomarkers that could aid in both prediction and intervention. Moreover, machine learning and precision medicine approaches should be increasingly applied not only to pharmacogenomic studies but also to refine diagnostic classification, considering potential subtypes across the SZ-AT spectrum, and personalize intervention strategies. Finally, future research should explore how to integrate digital phenotyping, real-time behavioral monitoring, and neurotechnological innovations that could provide dynamic, individualized insights into symptom trajectories and intervention outcome. Developing standardized, cross-disorder assessment tools and scalable interventions grounded in shared cognitive, genetic or neural differences holds promise for improving diagnostic precision and outcomes.

## References

[1] Oliver LD Moxon-Emre I Lai MC Grennan L Voineskos AN Ameis SH. Social cognitive performance in schizophrenia spectrum disorders compared with autism spectrum disorder: A systematic review, meta-analysis, and meta-regression. JAMA Psychiatry. (2021) 78 (3):281–92. doi: 10.1001/jamapsychiatry.2020.3908. American Medical Association., PMID: 33291141 PMC7724568

[2] Trevisan DA Foss-Feig JH Naples AJ Srihari V Anticevic A McPartland JC. Autism spectrum disorder and schizophrenia are better differentiated by positive symptoms than negative symptoms. Front Psychiatry. (2020) 11:548. doi: 10.3389/fpsyt.2020.00548, PMID: 32595540 PMC7301837

[3] Corbera S Wexler BE Bell MD Pittman B Pelphrey K Pearlson G. Disentangling negative and positive symptoms in schizophrenia and autism spectrum disorder. Schizophr Res. (2024) 271:1–8. doi: 10.1016/j.schres.2024.07.002, PMID: 39002525 PMC11384336

